# Nonketotic Hyperglycinemia: Insight into Current Therapies

**DOI:** 10.3390/jcm11113027

**Published:** 2022-05-27

**Authors:** Magdalena Nowak, Piotr Chuchra, Justyna Paprocka

**Affiliations:** 1 Students’ Scientific Society, Department of Pediatric Neurology, Faculty of Medical Sciences in Katowice, Medical University of Silesia, 40-752 Katowice, Poland; nmagdalena533@gmail.com (M.N.); piotr.chuchra456@gmail.com (P.C.); 2 Department of Pediatric Neurology, Faculty of Medical Sciences in Katowice, Medical University of Silesia, 40-752 Katowice, Poland

**Keywords:** nonketotic hyperglycinemia, inborn error of metabolism, treatment, sodium benzoate, NMDA antagonists, ketogenic diet, vagal nerve stimulation

## Abstract

Nonketotic hyperglycinemia (NKH) is a rare inborn error of glycine metabolism that is characterized by the accumulation of glycine in all tissues, especially in the central nervous system (CNS). Based on clinical outcomes, NKH can be divided into two forms, i.e., severe and attenuated NKH. A poor prognosis, including no developmental progress and intractable epilepsy, is typical of severe NKH, whereas patients with the attenuated form present with varied symptoms and neurodevelopmental outcomes. So far, no causal treatment of NKH is known. Currently, the therapy is based on sodium benzoate and NMDA (The N-methyl-D-aspartate receptor) receptor site antagonists (dextromethorphan, ketamine). Different clinical outcomes of the therapy raise doubts about the effectiveness of the treatment. The purpose of this review is to summarize the therapeutic potential, challenges and effectiveness of different NKH therapies.

## 1. Introduction

Nonketotic hyperglycinemia (NKH) is a rare neurometabolic disease caused by an inborn error of glycine metabolism, which results in the accumulation of glycine in all tissues, especially in the central nervous system (CNS) due to the disruption of glycine breakdown to carbon dioxide and ammonia [1]. The disease is inherited in an autosomal recessive manner. Mutations most often occur in the *GLDC* (9p22) and *AMT* (3p21.2) genes [2].

Based on clinical outcomes, NKH is divided into severe and attenuated NKH (a milder course). However, severe NKH is more prevalent and is characterized by more severe neurological symptoms such as no developmental progress, intractable epilepsy and apnea. Patients with attenuated NKH make variable developmental progress, present with treatable epilepsy or do not have epilepsy. In the neonatal period, a severe form of the disease is more common than attenuated NKH. The clinical presentation in the neonatal period includes lethargy progressing to coma and hypotonia, apnea and poor feeding with the first signs of the disease observed between the second week and third month of life. Patients may often develop both forms of NKH. Hypotonia is the most common symptom. Attenuated NKH is more often reported in patients that developed symptoms when over three months of age [1,3,4].

In patients with NKH, laboratory findings such as an isolated elevation of glycine levels in the plasma, cerebral spinal fluid (CSF) and abnormal CSF-to-plasma ratio (>0.08) are found. Additionally, characteristic brain changes may be seen in MRI [1,5]. The diagnosis is confirmed by molecular genetic tests [2,6].

So far, no causal treatment of NKH has been discovered, and no formal management guidelines have been developed for NKH. Currently, sodium benzoate and NMDA receptor site antagonists (dextromethorphan, ketamine) are used. Sodium benzoate can reduce the plasma glycine concentration, and NMDA receptor channel inhibitors can reduce overstimulation of NMDA receptors. Both drugs have different clinical outcomes [7,8,9]. This paper aims to present the therapeutic potential, challenges, effectiveness, advantages and disadvantages of different NKH therapies.

## 2. Predictors for Prognosis in Nonketotic Hyperglycinemia

Proper classification of NKH may be of particular importance for clinicians due to the differences in patients’ response to treatment, depending on the form of the disease. Adequate patient qualification can be particularly useful to predict benefits and side effects of the pharmacological treatment. It may also facilitate the decision to discontinue persistent therapy in patients who present from birth with a severe course of the disease and do not respond to treatment. Additionally, it enables a clinician to inform the patient’s family about a reliable prognosis [1,5].

The previous qualification of newborns and infants having severe NKH solely based on the early onset of symptoms led to an inadequate prognosis of the child’s development [5]. It may also have contributed to the various treatment efficacy results during the analysis of individual clinical cases [1]. This finding prompted a reassessment of the prognostic factors.

Currently, it is believed that the residual activity of the glycine cleavage enzyme system (GCS) is crucial for the development of a specific form of NKH, and it affects the clinical picture of the patient [7,10]. Patients with two alleles, without residual GCS activity, mostly present with severe NKH. However, each patient with at least one allele usually presents with a mild form of the disease with varying developmental potential [1,10]. The lack of residual enzyme activity leads to much greater glycine concentration, which may affect the effectiveness of drugs that lower the glycine level and may force the use of higher doses of drugs. It was demonstrated that the number of alleles with residual activity is related to the outcome. However, in a group of patients with attenuated NKH, no correlation was found between the clinical status specified as the achieved level of development and the cumulative activity of GCS [1,7]. Additionally, it was shown that the residual activity of GCS is necessary, although insufficient for the occurrence of an atypical form of NKH. This suggests that apart from the genotype, there are additional factors influencing the clinical picture [1].

Swanson MA et al. proposed the following factors as predictors of severe NKH: onset of symptoms in the first week of life (sensitivity = 87%, specificity = 100%), glycine concentration in CSF > 230 μM (sensitivity = 43%, specificity = 100%), the presence of a brain malformation in MRI (sensitivity = 71%, specificity = 92%), two non-missense mutations, two alleles leading to no residual activity expressed as the mutation score = −2 (sensitivity = 59%, specificity = 97%) and the glycine index > 3 (sensitivity = 87%, specificity = 100%) [1].

Attenuated NKH is diagnosed when the following are reported: manifestation of symptoms when over four months of age (sensitivity = 47%, specificity = 100%), CSF–plasma glycine ratio  ≤ 0.08 (sensitivity = 28%, specificity = 100%), no use of antiepileptic drugs (sensitivity = 70%, specificity = 100%), the presence of at least one allele ensuring residual activity expressed as the mutation score ≥ 1 (sensitivity = 76%, specificity = 94%) [1].

Moreover, it was suggested that predictors which are available at the early stage of diagnosis (age at onset ≥4 months, CSF glycine >230 μM, CSF–plasma glycine ratio ≤ 0.08, and brain malformations in MRI) may facilitate an adequate estimation of the outcome of the newborn (78% of patients with the severe form and 49% of patients with attenuated NKH) [1]. The disease is divided into severe and attenuated NKH based on reaching developmental outcomes expressed as the developmental quotient [1]. It should be emphasized that despite the correlation between a higher concentration of glycine in the CSF and the severe form of NKH, a low concentration of glycine does not rule out the occurrence of a severe form of NKH [5].

Due to the inability to predict the form of NKH based on glycine serum levels, Farris et al. developed the quantitative Weighted Multiparametric Mutation Score (WMMS). It was created based on retrospective analyses of patient records. Firstly, the researchers used predictions of protein stability, interaction models and evolutionary conservation to create the Multiparametric Mutation Score (MMS) that can be applied to assess 251 of 255 existing mutations. Secondly, the researchers created a quantitative scale of clinical disease severity that chooses four major disease domains that are present in more than 30% of the patients (cognitive disorders, seizure, muscular and motor control and brain malformation). For each domain, a scale of 0 to 3 points with a maximum score of 12 points obtained by the patient was used. The sum of four domain points was termed the “clinical outcome score” (COS). It was used for the optimization of MMS, creating the WMMS. Based on the WMMS, it is possible to distinguish with high accuracy between the severe and attenuated type (*p*-value of 1.2 × 10^−5^ for homozygous patients and *p*-value of 3.5 × 10^−7^ for heterozygous patients) [4].

Among a wide spectrum of useful diagnostic tests, the special value of magnetic resonance imaging should be emphasized in the assessment of patient prognosis.

Hennermann et al. proved that the presence of severe malformations, including the most frequently diagnosed agenesis of the corpus callosum, strongly correlated with the occurrence of a severe form of the disease. Therefore, MRI imaging should be performed as soon as possible [5]. The correlation between the corpus callosum image and the clinical phenotype indicates the importance of this factor as one of the first objective indicators, which allows a prediction of severe NKH in newborns with a sensitivity of 60–70% [11]. In infancy, the predictive value is much greater. Stence et al. observed that after one month of age, none of the patients with severe NKH had a normal image of the corpus callosum [11]. On the contrary, the importance of EEG as a prognostic factor has a much lower predictive value, since the characteristic changes in EEG, such as EEG patterns with hypsarrhythmia or a burst-suppression-pattern, which are frequent in the severe form, occur also in attenuated NKH [5,12].

## 3. Pharmacotherapy

Currently, there is no treatment standard for patients with NKH, and no effective causative treatment is known. For many years, the therapy has been mainly focused on reducing plasma glycine levels with sodium benzoate and the use of N-methyl D-aspartate receptor (NMDA) antagonists, such as dextromethorphan or ketamine [13,14,15]. Attempts to use the above-mentioned therapy resulted in a different clinical response in patients or a complete lack of response, which raises doubts as to the validity of the therapy. In some patients with the classical variant of NKH, no clinical improvement was observed despite the early use of sodium benzoate and/or dextromethorphan, even if a reduction in glycine plasma concentration was temporarily achieved [3,16,17]. The wide variation in therapeutic effects may be influenced by the heterogeneity of NKH-inducing mutations, which has not always been considered [8].

Sodium benzoate significantly lowers the plasma concentration of glycine and decreases the concentration of glycine in the CSF, but not to normal values [14,18]. The therapeutic goal is to achieve a plasma concentration of 120 to 260 µmol/L in a sample taken one to two hours after drug administration [15]. The metabolism of sodium benzoate depends on the available glycine pool, which is conditioned by the activity of the glycine cleavage enzyme system. Depending on the form of the disease, there are differences in the effective dose amount of sodium benzoate [15,19]. Patients with a severe form of the disease and a correspondingly higher glycine index require higher doses of sodium benzoate (550–750 mg/kg/day). In patients with the attenuated form of the disease and a lower glycine index, lower doses of sodium benzoate (200–500 mg/kg/day) should be used. Research suggests that sodium benzoate therapy should be started with the lowest dose recommended for the specific form of the disease. If the therapeutic goal is not achieved, the dose should be increased by 50 mg/kg per day until the target dose is reached. Glycine and benzoate concentrations should be controlled before a change of the dosage [15]. It is very important to regularly monitor the glycine index during treatment with sodium benzoate during a change in drug dosage and due to the dietary factor [15].

Based on the follow-up of patients with NKH, it was shown that the use of sodium benzoate therapy reduced the frequency of epileptic seizures and improved patient alertness [14,15,20,21,22,23,24]. However, in severe disease, it did not affect the progress of psychomotor development or drug-resistant epilepsy. Due to the fact that the implementation of the therapy did not prevent the occurrence of a serious delay in psychomotor development, the therapy was considered ineffective [8,9,20].

Hennermann et al. observed that the use of sodium benzoate had a positive effect in both initial and long-term treatment in most cases of NKH. This observation was seen in all variants of NKH. The positive effect of treatment was defined as a reduction in the plasma glycine level to ≤300 μmol/L as a biochemical parameter, and a positive clinical effect of treatment was expressed as an increase in alertness, reduction in seizure frequency or a decreased number of anticonvulsants. The positive effect of benzoate therapy was achieved in 22 of 26 patients with severe neonatal NKH, six of six patients with neonatal attenuated NKH, two of three patients with severe infantile NKH and one of three patients presented with attenuated infantile NKH [5]. However, it should be emphasized that the effectiveness of the therapy, the impact on the psychomotor development of the patients and the attainment of the milestones were not assessed. Moreover, it was suggested that the lack of effectiveness of sodium benzoate therapy in some patients could be caused by the use of too-low drug doses. In that study group, children who did not achieve a positive effect of treatment had a daily dose of sodium benzoate below 290 mg/kg [5]. This may be due to the fact that the doses of sodium benzoate required to effectively lower the glycine concentration differ depending on the form of the disease [5,15]. Poothrikovil et al. reported an improvement in a patient’s clinical condition only 24 h after sodium benzoate administration with a reduction in the glycine level by 50% and a mild improvement on the Glasgow Coma Scale [25].

Several side effects should be considered before deciding whether to initiate treatment or to increase the dose of sodium benzoate. The use of sodium benzoate (even in therapeutic doses) is associated with the risk of gastrointestinal complaints, such as gastritis, gastroesophageal reflux and esophagitis. Renal tubular dysfunction can be another treatment-related side effect [5,6,14,15,18,26]. Carnitine deficiency may occur as another adverse effect of sodium benzoate therapy [15,23]. Exceeding the maximum dose may result in the symptoms of sodium benzoate poisoning, including coma, metabolic acidosis and a number of electrolyte disturbances, e.g., hypokalemia and hypocalcemia [15]. Blood levels of glycine, sodium benzoate and carnitine should be monitored regularly to minimize the risk of adverse effects. Additionally, the drug can be used in the form of coated benzoate granules [5].

Abnormal levels of glycine in NKH may result in excessive stimulation of NMDA receptors, which is suggested to be involved in the pathomechanism of the disease, especially in early brain damage [27]. Dextromethorphan and ketamine are the most common NMDA antagonists. Dextromethorphan can be used in four doses (5–35 mg/kg/day). It is possible to monitor the drug concentration, but the therapeutic level is not defined. It is suggested to maintain drug concentration levels above 0 and below 100 nmol/L [28].

It has been suggested that NMDA antagonists may lead to reduction in seizures, normalization of the EEG recording and may also positively influence muscle hypotonia and apnea [8,14,29,30]. Iqbal et al. reported a case of a patient diagnosed with a variant between the classical neonatal and infantile form of NKH. NMDA antagonist therapy (dextromethorphan) and a ketogenic diet (KD) were used, which resulted in decreased seizure frequency and an improvement in alertness. However, the patient had poorly controlled epilepsy and global developmental delay in long-term treatment [30]. Boneh et al. observed better clinical outcomes in two patients treated early with sodium benzoate and an NMDA antagonist (ketamine) compared to those of three patients who were administered sodium benzoate and non-NMDA modulators. The patients on NMDA therapy obtained not only improvements in hypotonia, apnea and respiration abnormalities, but also a proper level of alertness and the attainment of milestones with no history of seizures. They suggested that there could be a correlation between an early introduction of NMDA antagonist therapy and a positive neurological outcome, emphasizing the superiority of ketamine over other NMDA antagonists [29]. However, there are several side effects of ketamine therapy, including increased sleepiness, over-agitation or involuntary movements [18].

Confirming previous observations, Hennermann et al. demonstrated that dextromethorphan initially resulted in a good clinical response in all variants of NKH, but long-term therapy was effective mostly in children with attenuated NKH. The positive effect was expressed as an increase in alertness, a decrease in seizures or a decreased number of anticonvulsants. The positive initial effect of treatment was achieved in 8 of 13 patients with severe neonatal NKH, two of two patients with attenuated neonatal NKH, one of two patients with a severe infantile form and three of three patients with attenuated infantile NKH. However, an effect of long-term therapy was observed only in 3 of 11 patients with severe neonatal NKH. This observation led to the conclusion that dextromethorphan could be more effective during long-term treatment in attenuated rather than in severe NKH [5]. They also observed an improvement in respiration during the neonatal period in all NKH variants after strychnine administration. However, the long-term use was limited due to adverse effects [5].

Combination therapy with sodium benzoate and NMDA antagonists is the most commonly used therapy in the treatment of NKH patients. Response to this therapy and clinical outcomes show significant differences between patients with severe and attenuated NKH.

The immediate introduction of treatment in severe NKH can modify the early phase of the disease, which results in the prevention of apnea and severe hypotonia. However, therapy does not contribute to seizure prevention or developmental delay [8,22,31]. This finding was shown by Korman et al. in a child treated from birth with sodium benzoate (250 mg/kg/day) and ketamine (15 mg/kg/day). Therapy resulted in a benign neonatal course with no hypotonia or apnea, which occurred in the nontreated sibling also affected with NKH. However, long-term follow-up showed the occurrence of myoclonic jerks, minimal responsiveness and severe psychomotor developmental delay despite the continuation of treatment [8].

Similarly, Shin et al. also showed initial improvement of patient condition expressed as an improvement of apnea and better seizure control after an introduction of sodium benzoate and dextromethorphan therapy in the first days of life. However, the future neurological outcome was poor with continuous, intractable seizures, and no milestones were attained [31].

Ichikawa et al. reported a patient treated with high-dose benzoate (600 mg/kg) and dextromethorphan (20 mg/kg) from 14 days of age. During the neonatal period, the following were reported: amelioration of encephalopathic symptoms, increased alertness, decreased seizures and a reduction in glycine levels in the CSF. However, in long-term therapy, the patient developed severe psychomotor impairment and refractory seizures with glycine plasma levels >300 μmol/L despite the high-dose benzoate treatment [22].

Different therapy outcomes were achieved by Poothrikovil et al. Initially, they observed improvement in the prevalence of myoclonic jerks and hypotonia after early administration of sodium benzoate and ketamine [25]. They noted that together with treatment progress and clinical improvement of their patient, there was also improvement in EEG findings. However, a continued burst-suppression pattern was still present, even after the positive clinical effect of the therapy and after the normalization of glycine in the plasma. However, they observed no clinical improvement of this therapy in another patient with neonatal severe NKH. Despite drug administration from eight weeks of life, the patient presented with status epilepticus, alertness disturbances, and in long-term follow-up, the patient did not attain developmental milestones, and no clinical improvement was noticed. This suggests about ongoing pathophysiologic mechanisms of the irreversible brain injury that is unresponsive to current therapy [25].

Highlighting the diversity of the effectiveness in NKH variants, Bayrak et al. showed that therapy with sodium benzoate (200–500 mg/kg/day) and dextromethorphan (5–10 mg/kg/day) resulted in are duction in seizure frequency and an improvement in alertness. However, only one patient with infantile attenuated NKH was seizure-free and made developmental progress. Another patient had a poor prognosis with severe developmental delay. The therapy in severe NKH influenced only partial seizure control and the normalization in the glycine level with no impact on the developmental outcome [20].

The findings of the studies above confirm that therapy with benzoate and dextromethorphan has a positive effect during the neonatal period. It results in a significantly milder course of severe NKH in the neonatal period, which includes improvement in apnea, respiratory disturbances, muscle hypotonia or a reduction in seizure frequency [8,18,20,25,31]. It may have a positive effect on the quality of life of the patients with the severe variant of the disease. Nevertheless, the efficacy of long-term therapy in severe NKH is limited. Despite continued treatment, the condition gradually deteriorates, and the typical symptoms of severe NKH occur, i.e., no progress in psychomotor development and drug-resistant epilepsy [8,18,20,25,31]. Unsatisfactory long-term treatment results may be due to prenatal brain damage, which suggests the exposure of the fetus to high levels of glycine in severe NKH [8].

Different effects of sodium benzoate and NMDA antagonist therapy were observed in patients with attenuated NKH. It was shown that in patients with attenuated NKH, the therapy improves the EEG recording and results in the discontinuation or significant reduction in epileptic seizures and progression of psychomotor development [5,7,24].

Brunel-Guitton et al. observed that in patients with late manifestation of NKH, the addition of dextromethorphan to sodium benzoate therapy could have a positive effect on the reduction in myoclonic seizures [32]. Moreover, Suzuki et al. suggested that better seizure control in patients could be achieved by switching dextromethorphan to high doses of ketamine [33].

Studies on siblings showed that the optimal therapeutic effect in children with attenuated NKH is influenced by the earliest administration of sodium benzoate and dextromethorphan and the continuation of therapy in the early stages of child development [7,34].

Bjoraker et al. compared pairs of siblings with attenuated NKH. The first child was treated after two to six months, while the second child was diagnosed prenatally or neonatally and was treated effectively from the first week with benzoate sufficient to control glycine levels (<400 μM) and dextromethorphan (≥3 mg/kg/d) for at least the first two years. The children who were treated from birth achieved better neurodevelopmental outcomes, attained milestones earlier and acquired more skills than the first sibling did. The improvement in the clinical outcome was also evident in cognitive abilities (expressed as a higher IQ or GDQ level), communication skills and adaptive skills (measured in Vineland Adaptive Behavior Scales) in children treated from the neonatal period. Moreover, the children treated from birth presented with less frequent and less severe seizures or the absence of seizures compared with those of other siblings. However, the therapy did not affect the symptoms characteristic of the attenuated form, i.e., hyperactivity and chorea [7].

In children diagnosed with attenuated NKH, treatment with sodium benzoate and an NMDA antagonist should be introduced immediately during the first years of life to provide the optimal neurodevelopmental outcome. Based on the positive effect of the therapy, it is suggested that treatment may be a modifying factor of the genotype–phenotype relationship in children with attenuated NKH [7].

Treatment with sodium benzoate is beneficial both in the initial phase and in long-term treatment. On the other hand, the use of dextromethorphan, despite its initial beneficial effect in the treatment of all forms of NKH, is beneficial mainly for patients with an atypical form of the disease in the long term [5]. Moreover, NMDA antagonists should be administered as soon as possible to improve neurological status [8,29,34].

An attempt to use perampanel, an antagonist of AMPA receptors, was reported in a patient for whom treatment with sodium benzoate and dextromethorphan was unsuccessful. By gradually increasing the dose to 0.65 mg/kg/day, an epileptic spasm reduction of50% was achieved [17].

Exploratory treatment with cinnamic acid was proposed as another strategy to lower glycine accumulation. Cinnamic acid is an intermediate in the glycine conjugation pathway. It was suspected to act in a manner similar to that of benzoate in the NKH mouse model. It was proven that the administration of sodium cinnamate to *GLDC*-deficient mice resulted in a reduction in the plasma glycine concentration with a dose-dependent effect and led to significantly lower abundance of glycine in the liver and brain tissue [35]. Further research confirming these results should be conducted to provide information on whether cinnamate can be used in the treatment of NKH patients. Additionally, the effects of cinnamate-based therapy should be compared with the results of sodium benzoate therapy.

There are no official recommendations regarding the duration of pharmacotherapy in NKH. Withdrawal of therapy may have a variety of clinical effects.

The attempts above to reduce the doses and finally discontinue the use of sodium benzoate resulted in relapse or worsening of existing symptoms [24,31]. Withdrawal of benzoate therapy in an infant resulted in the redevelopment of frequent seizures, apnea and an increase in glycine levels in the plasma and CSF. After the re-introduction of the treatment, the clinical condition improved [31]. Withdrawal of sodium benzoate resulted in behavioral changes and epileptic activity in the EEG [24].

Moreover, after discontinuation of dextromethorphan therapy, significant clinical deterioration coinciding with epilepsy and slow EEG high-voltage activity was reported. After reintroducing dextromethorphan, recovery was achieved within 24 h [36]. Contrary to these observations, no changes in a patient’s clinical picture or EEG recording were reported following withdrawal of dextromethorphan after one year of administration in attenuated NKH [24]. Hamosh et al. observed no clinical changes after withdrawal of dextromethorphan in a five-year-old patient [37].

In addition, there were reports of patients whose symptoms did not recur despite permanent treatment discontinuation. Korman et al. described patients with mild attenuated NKH, in whom discontinuation of therapy (at two and four years of age) with both sodium benzoate and ketamine did not worsen neurological symptoms also at later follow-up visits [34]. This suggests that the effect of withdrawal may be influenced by the form of the disease, patient age and treatment duration.

Another form of glycine-lowering therapy is the reduction in glycine intake using low-glycine diets, including protein parenteral amino-acid-free nutrition and a glycine-free formula [26,31,32]. In patients with severe disease who require high doses of sodium benzoate, the introduction of a glycine-restricting diet supports the maintenance of an appropriate glycine index [15]. Such a dietary restriction is not necessary for patients with attenuated NKH, whose glycine levels are controlled by sodium benzoate. However, after a low-glycine diet, improvement in symptom control, including hyperactivity and myoclonic seizures, was observed in a patient with attenuated NKH [32]. A glycine-restricted diet alone is not sufficient to achieve a therapeutic effect [38].

## 4. Antiepileptic Drugs (AEDs)

Treatment of epileptic seizures is an integral part of the therapy of NKH. A particular challenge is the treatment of patients with the classic form of the disease in whom drug-resistant epilepsy is the most common [1]. These patients are given, on average, three or even four AEDs as opposed to patients with attenuated NKH, in whom often only a single drug is required to control seizures [1,5]. Moreover, it has been shown that mutations in the *AMT* gene require more antiepileptic drugs more often than mutations in the *GLDC* gene do [39].

Due to the heterogeneous nature of epileptic seizures, a variety of AEDs are used in NKH. Hennermann et al. showed effectiveness of phenobarbital in the treatment of epileptic seizures during the neonatal period with a positive effect of therapy in 18 of 34 patients. Partial seizure control was achieved with the following drugs: primidone (4/5), levetiracetam (2/3), phenytoin (2/9), vigabatrin (3/14), topiramate (1/4), carbamazepine (1/5) and clobazam (1/6). The use of felbamate, an NMDA receptor blocker, in managing drug-resistant seizures is limited by side effects. However, no effects were obtained during treatment with lamotrigine and sulthiame [5].

Valproate (VPA) should be avoided in patients with NKH due to its inhibitory effect on GCS activity, which enhances glycine accumulation [21]. Treatment with VPA may result in a significant increase in the frequency and number of epileptic seizures, which can be observed when the dose of the drug is increased [21,40].

Some patients, especially those with severe disease, fail to achieve seizure control despite the use of many AEDs. In these cases, it may be beneficial to consider nonpharmacological treatment of seizures [16].

## 5. Nonpharmacological Treatment of Epileptic Seizures

The ketogenic diet (KD) is successfully used in pediatric patients as a nonpharmacological treatment for drug-resistant epilepsy of various etiologies, including seizures in metabolic diseases [12,41]. In NKH patients, especially those with severe disease, KD may be of particular benefit because of the presence of drug-resistant epilepsy, which is related to this variant of the disease [16,26].

Cusmai et al. observed a beneficial effect of a KD in three patients with severe NKH with early myoclonic encephalopathy. In these patients, seizure control was not achieved despite the use of sodium benzoate and NMDA antagonists with multiple antiepileptic drugs. The addition of a KD to the standard therapy led to a significant reduction in various epileptic seizures (myoclonic jerks, tonic spasms, partial seizures), achieving a reduction in seizures of over 50% compared to the frequency before the introduction of the KD [16]. Similarly, Kava et al. achieved a significant reduction in seizure frequency and in antiepileptic drug use in a patient with epileptic encephalopathy with several seizure types, including flexor spasms, multifocal clonic and myoclonic seizures [26]. In addition, lowering glycine levels, improving tonic spasm and tonic-clonic seizure control and complete resolution of tonic-clonic seizures when the KD was used were also reported [3]. It has been shown that the use of this diet can bring benefits even in NKH patients with very severe forms of seizures, including the resolution of super-refractory status epilepticus (SRSE) [42]. It was also reported that the use of dextromethorphan and a KD did not prevent the development of uncontrolled epilepsy despite an initial improvement in seizure control [30].

The clinical effect of the implemented therapy was found in some patients as early as in the first week of treatment [16,26]. It has also been found that seizure reduction is maintained during continued long-term use of the diet [6,16,26].

Moreover, it has been shown that a KD also resulted in increased alertness, improvement of muscle tone and reduction in spasticity. Decreased plasma and CSF glycine levels in patients following the KD indicated that this diet could be used as a therapy supporting the reduction in glycine levels [6,16,26]. The diet was generally well-tolerated by patients. However, problems with glycemic control and body weight were reported [6,16,26,42].

It should be emphasized that the diet does not affect the development of severe developmental delay, which is a typical complication of severe NKH.

The KD significantly increases the comfort of life and reduces the number of hospitalizations by improving the control of epileptic seizures. Some patients reported a complete reduction in epileptic seizures or a reduction in the number of AEDs [3,16,26]. This may be of particular importance to the families of patients with severe NKH, for whom therapeutic strategies that affect the quality of life are severely limited.

Vagal nerve stimulation is another nonpharmacological therapeutic option that may be offered to patients with severe NKH. A few reports on the use of this method in NKH indicate a beneficial effect in patients who have failed to achieve seizure control despite the use of glycine-lowering therapy, NMDA antagonists and multiple AEDs [3,43,44].

Tsao et al. described two patients with intractable seizures associated with severe NKH who were treated with standard therapy and multiple anticonvulsants. Implantation of vagus nerve stimulation in the first patient resulted in the reduction in the prevalence of myoclonic and tonic seizures of>75%. Additionally, the number of antiepileptic drugs was decreased from six to three. Another patient with persistent tonic seizures gained a reduction in seizure frequency of90% and total control of seizures in long-term vagus nerve stimulation therapy with discontinuation of all anticonvulsants [44]. The positive effect of vagal nerve stimulation was also observed in a patient with status epilepticus with a reduction in seizure frequency of over 90% and a dose reduction of AEDs. Moreover, the patient did not develop status epilepticus again [43]. Decreased seizure frequency was also obtained in a patient with infantile spasms. However, the seizures were not fully controlled [3].

It should be emphasized that the effect of vagal nerve stimulation leads to an increase in the quality of life and improvement of alertness due to better seizure control and are duction of multiple unnecessary anticonvulsants [43,44]

The effectiveness of different forms of therapy in NKH is briefly summarized in Table 1.

## 6. Complications and Multidisciplinary Care

Patients affected with NKH require multidisciplinary care due to a number of complications resulting from disease progression or treatment.

Constant gastroenterological care may be necessary due to the occurrence of disorders such as feeding problems requiring the use of a gastric tube, gastroesophageal reflux, esophagitis and gallstone disease. These disorders are reported more often in patients with severe NKH [5].

NKH patients often require cardiac control from birth, including echocardiography due to possible congenital anomalies such as mitral valve defects, patent ductus arteriosus, patent foramen ovale or dilated cardiomyopathy [20,45]. A possible link between NKH and pulmonary hypertensive vascular disease (PHVD) should be emphasized. In some patients, the development of pulmonary hypertension may be the first symptom of NKH, even without accompanying neurological symptoms. Therefore, an appropriate metabolic assessment should be performed in all patients with an unexplained PHVD background. This is especially important when a pneumothorax develops after vasodilatation, even without accompanying neurological symptoms [46].

Another complication of NKH is the occurrence of musculoskeletal disorders, resulting from CNS dysfunction [4,47]. Severe multiple joint contractures that limit mobility or progressive neuromuscular scoliosis of early onset and painful dislocations of the hip joints are particularly common. Patients with orthopedic disorders are also at risk of developing thoracic failure syndrome [47].

Considering the critical clinical condition and a poor developmental prognosis, despite the use of optimal therapy in patients with severe disease, withdrawal of futile medical care may be an acceptable decision for the patients’ families. This mainly applies to patients with respiratory failure and apnea, which requires the use of a ventilator and also to patients with severe brain malformations [30,48].

## 7. Conclusions

NKH is a rare neurometabolic disease with no effective causal treatment. Due to diverse clinical responses to therapy with sodium benzoate and NMDA antagonists, the justification for long-term treatment has been questioned. In this review, we highlighted that the effectiveness of this treatment varies depending on the form of the disease. This indicates the need for the appropriate classification of patients not only based on the age of symptom onset but also other factors, e.g., genetic mutations or the presence of cerebral anomalies. Moreover, in both forms of the disease, the benefits of pharmacotherapy are observed. However, in severe NKH, benefits of the therapy are limited to a decrease in symptom severity in the neonatal period without preventing the development of subsequent complications of NKH such as developmental delay and drug-resistant epilepsy. On the contrary, in attenuated NKH, implementation of treatment results in an improvement in neurodevelopmental outcomes. Early treatment initiation may be crucial in both forms of the disease. Patients with severe NKH and drug-resistant epilepsy may benefit from nonpharmacological treatment such as a ketogenic diet or vagal nerve stimulation. Further studies are warranted on larger groups of patients to provide more detailed information about the optimal duration of the therapy, differences between particular drugs and the effectiveness of nonpharmacological epilepsy treatment.

## Figures and Tables

**Table 1:** Effectiveness of different therapies in NKH.

Therapy	Clinical Outcome	Patients Group	Authors
Sodium benzoate	Reduction in plasma glycine level to ≤300 μmol/L, increase in alertness, decrease in seizures or a decreased number of anticonvulsants.	22/26 with severe neonatal NKH, 6/6 with attenuated neonatal NKH, 2/3 with severe infantile NKH, 1/3 with attenuated infantile NKH.	Hennermann et al. [5]
Dextromethorphan	Increase in alertness, a decrease in seizures or a decreased number of anticonvulsants.	8/13 with severe neonatal NKH, 2/2 with attenuated neonatal NKH, 1/2 patients with severe infantile form, and 3/3 patients with attenuated infantile NKH.	Hennermann et al. [5]
Sodium benzoate and dextromethorphan	Improvement in apnea and initially better seizure control.	Patient with neonatal NKH.	Shin et al. [31]
	Amelioration of encephalopathic symptoms, increased alertness and decreased seizures (temporary) and (temporary) reduction in CSF glycine levels.	Patient with neonatal NKH.	Ichikawa et al. [22]
	Reduction in seizure frequency and an improvement of alertness, developmental progress (only patients with attenuated NKH).	9 patients with severe NKH and 1 patient with attenuated NKH.	Bayrak et al. [20]
	Improvement in neurodevelopmental outcome and seizure control.	4 patients with attenuated NKH.	Bjoraker et al. [7]
	Reduction in myoclonic seizures.	Patient with attenuated NKH.	Brunel-Guitton et al. [32]
Sodium benzoate and ketamine	Benign neonatal course with no hypotonia and apnea.	Patient with severe NKH.	Korman et al. [8]
	Improvement in the frequency of myoclonic jerks and hypotonia.	1 of 2 patients with severe NKH.	Poothrikovil et al. [25]
	Improvement in hypotonia, apnea and respiration abnormalities, the proper level of alertness and attainment of developmental milestones.	2 patients with neonatal NKH.	Boneh et al. [29]
Perampanel	Reduction of 50% of epileptic spasms.	Patient with severe NKH.	Daida et al. [17]
Ketogenic diet	Reduction of over 50% of seizures.	3 patients with severe NKH with early myoclonic encephalopathy.	Cusmai et al. [16]
	Reduction in seizure frequency and antiepileptic drugs, spasticity reduction.	Patient with severe NKH.	Kava et al. [26]
	Lowering glycine levels, improvement of tonic spasms and tonic-clonic seizure control and complete resolution of tonic-clonic seizures.	2 patients with severe NKH.	Shbarou et al. [3]
	Resolution of super-refractory status epilepticus (SRSE).	Patient with severe NKH.	Appavu et al. [42]
Vagal nerve stimulation	Reduction of >75% of myoclonic and tonic seizure frequency, reduction in tonic seizures (of 90%), withdrawal or reduction of antiepileptic drugs from 6 to 3.	2 patients with severe NKH.	Tsao et al. [44]
	Reduction in seizure frequency of more than 90%, dose reduction of AEDs.	Patient with neonatal NKH.	Grioni et al. [43]

## Data Availability

Not applicable.
